# Contest of Best Practices tackling social inequalities in cancer prevention: an iPAAC initiative

**DOI:** 10.1093/eurpub/ckab206

**Published:** 2021-12-15

**Authors:** Marta Hernández-García, Dolores Salas-Trejo, Ahti Anttila, Satu Lipponen, Ana Molina-Barceló

**Affiliations:** 1 Fundación para el Fomento de la Investigación Sanitaria y Biomédica de la Comunitat Valenciana, FISABIO, Valencia, Spain; 2 Finnish Cancer Registry, Cancer Society of Finland, Helsinki, Finland; 3 Cancer Society of Finland, Helsinki, Finland

## Abstract

Current health promotion and early cancer detection programmes yield different results depending on the social group and have a different impact among individuals. Thus, they may generate social inequalities in health. The Contest of Best Practices tackling social inequalities in cancer prevention is an initiative that emerged in the framework of the Innovative Partnership for Action Against Cancer Joint Action. This contest identifies interventions that have proven to be effective in reducing social inequalities in cancer prevention in European countries, with the aim of sharing lessons learned and inspiring solutions, as well as facilitating replication in other health systems and similar social settings.

## Introduction

Many cancer risk and protection factors such as tobacco consumption, diet, exercise and cancer screening have socially determined conditions. In general, people who pertain to lower socioeconomic groups are more exposed to risk factors and less exposed to protective ones. Consequently, socially disadvantaged groups are at higher risk of most common cancers.^1^ Successful cancer prevention requires a public health approach with targeted actions for deprived groups, directing interventions at the population as a whole and with additional emphasis on vulnerable groups.^2^

The European Commission’s Third Health Programme states that in order to promote health, prevent diseases and foster supportive environments for healthy lifestyles, good practices should be identified and their uptake promoted, in particular addressing key lifestyle-related risk factors with a focus on EU added value.^3^ Documenting and sharing ‘Best Practices’ (BPs) provide information on lessons learned and insight to improve strategies, with the aim of implementing larger-scale, sustained and more effective interventions.^4^

In this light, the Contest of BPs tackling social inequalities in cancer prevention was organized in the framework of the Innovative Partnership for Action Against Cancer (iPAAC) Joint Action, co-funded by the EU’s Third Health Programme. This contest is aimed at identifying and compiling relevant European experiences and contributing to the dissemination of cancer prevention BPs from a social standpoint.

## Methods

According to national and international organizations,^4–6^ the term BP is defined as an innovative and relevant intervention or managerial model implemented in a real-life setting, which has been favourably assessed in terms of adequacy, equity, effectiveness and efficiency.

As a preliminary step, a ‘Call for Experts’ was organized to select independent experts to assess the submitted proposals. Candidates were evaluated by the Contest Management Board, according to expertise on epidemiology, public health and social disciplines; ensuring objectivity and avoiding conflicts of interest. The BP Contest evaluation board was composed of the independent experts selected in the ‘Call for Experts’, and two members of the Contest Management Board. Each reviewer was provided with assessment guidelines, including references and explanations of evaluation criteria.

The contest was launched on the iPAAC website (www.ipaac.eu); the guide and application form were available online. The submitters’ guide detailed the rules of participation and evaluation criteria, while the application form contained information on fulfilment of the criteria, a description of the intervention and a self-assessment.

The proposals were assessed on a peer-review basis. In order to be accepted for evaluation, practices had to meet several compulsory criteria: relevance, equity and effectiveness. Any proposals that failed to fulfil these requirements were excluded. Interventions were further assessed in terms of gender perspective, efficiency, ethics, sustainability, inter-sectorial collaboration, transferability, innovation, evidence/theory based and public engagement. Each criterion was assessed on a scale from 0 to 5 according to specific definitions. Proposals achieving a score of 27 points or higher were deemed to be a ‘BP’.

Personal data were processed in accordance with Regulation 2016/679 of the European Parliament and of the Council on personal data protection. Experts received no fees for their contribution and declared no conflicts of interest.

## Results

Overall, 15 proposals were submitted, coming from Belgium (1), France (5), the UK (2), Italy (1), Slovenia (1) and Spain (5). Six of the 15 proposals (40%) fell under the scope of health promotion and 8 (53%) addressed cancer screening. One practice (7%) approached both primary and secondary preventions. Eleven proposals (73%) were considered BPs (table 1).

**Table 1:** Summary of acknowledged best practices, topic category, promoting institution and country

Topic	Title	Organization (country)
Cancer screening	Improving informing decision making in the Flemish cancer screening programmes for persons with a disability	Centre for Cancer Detection (Belgium)
Cervical cancer screening	GP-endorsed cervical screening text reminders in London	England/Improvement/Public Health England (UK)
Colorectal cancer screening	Effects of evidence-based strategies to reduce the socioeconomic gradient of uptake in the English NHS bowel cancer screening programme	Public Health England (UK)
Colorectal cancer screening.	Primary care involvement as a key to reduce inequalities in the colorectal cancer screening	Basque Country Regional Ministry of Health (Spain)
Colorectal cancer screening	Slovenian national colorectal cancer screening—SVIT Programme	National Institute of Public Health (Slovenia)
Health promotion and cancer screening	Bringing cancer prevention closer to the most vulnerable population	Alzira Local Centre for Public Health (Spain)
Health promotion—Diet, nutrition	Nutri-Score	Nutritional epidemiology research team—Paris 13 University (France)
Health promotion—Diet, nutrition	OPTICOURSES programme, participatory workshops (demand side)	French National Institute for Agricultural Research, INRA (France)
Health promotion—Diet, nutrition and physical activity	Vivons en Forme (Let’s live healthy) programme	Fédérons les villes pour la Santé-FLVS (France)
Health promotion—Physical activity	Programme for prescribing health assets for physical activity	Public Health Directorate. Valencia Regional Ministry of Health (Spain)
Health promotion—Tobacco	TABADO	French National Cancer Institute (France)

Healthy diet was the main protection factor addressed by the health promotion intervention BPs (3/6 BP; 50%), tobacco (1/6; 17%) and physical activity/body weight (2/6; 33%) were at the core of several proposals. Primary prevention BPs included ‘NutriScore’, which provides overall nutritional quality information so that consumers can make healthier choices; ‘Opticourses’, which improves the food quality–price ratio for deprived populations; ‘Vivons en forme’, which aims to prevent obesity in children and reduce social inequalities by promoting a healthy lifestyle among disadvantaged families; ‘Health assets for physical activity’, which prescribes physical activity; ‘Tabado’, which evaluates the transferability of a smoking cessation programme aimed at students in vocational training centres; and ‘Bringing cancer prevention closer to the most vulnerable population’, which promotes favourable attitudes towards cancer prevention among deprived populations.

As for secondary prevention, practices were mainly focused on bowel cancer screening programmes (3/6; 50%), whereas several interventions addressed cervical cancer screening (1/6; 17%) or simultaneously focused on different programmes (2/6; 33%). Secondary prevention BPs comprised ‘Improving informed decision making in the Flemish cancer screening programs for persons with a disability’, which improves the digital accessibility of screening information; ‘General practitioner-endorsed cervical screening text reminders in London’, which reduces age inequalities in screening uptake; ‘Effects of evidence-based strategies to reduce the socioeconomic gradient of uptake in the English NHS bowel cancer screening programme’, which decreases the socioeconomic gradient in screening uptake; ‘Primary care involvement as a key to reduce inequalities in colorectal cancer screening’, which involves primary care staff in order to increase participation rates and decrease inequalities; and ‘Slovenian national colorectal cancer screening—SVIT Programme’, which increases the participation of people with a lower educational level, the male population and communities with the lowest response.

Readers can find out more about the selected BPs by visiting www.ipaac.eu/en/contest-best-practices/.

## Discussion

This contest made it possible to identify health and social interventions that reduce inequalities in cancer prevention, providing an insight on effective strategies that can be translated to other settings and adapted into new, more equitable procedures.

As in previous initiatives for documenting and sharing BPs,^4–6^ the submitted proposals were assessed according to rigorous criteria: effectiveness, relevance, efficiency, ethics, sustainability, transferability, inter-sectorial collaboration and public engagement. However, equity is an inherently major issue here, which is why, unlike previous actions, it is considered a compulsory criterion. This feature adds value to this contest and makes it different and innovative compared to endeavours led by the World Health Organization,^4^ the European Commission Directorate-General for Health and Food Safety,^5^ or the Spanish Ministry of Health.^6^

Some of the interventions are similar to the results found in recently published systematic reviews. Regarding tobacco consumption, a review performed by Smith *et al.*^7^ showed that behavioural counselling delivered in a community setting and tailored to individual needs appeared to demonstrate a positive impact on smoking cessation outcomes among older, deprived smokers, similar to the result we found for teenagers in vocational training centres. Finally, Bygrave *et al.*^8^ also found that interventions based on simplified screening information leaflets, as well as general practitioner-endorsed invitations, improved equity in cancer screening programme attendance.

No proposals related to patient navigator programmes (providing guidance for cancer care by trained professionals) were received. Patient navigation has proven to be effective in reducing cancer inequalities, showing improved screening rates among deprived groups in high-income countries.^9^

In conclusion, these results suggest that a combination of universal public health interventions together with targeted interventions may be more effective in preventing cancer in the population as a whole, integrating specific actions for certain groups that are not otherwise reached by universal prevention activities. Tackling inequities should be included in future BP criteria, as well as future editions of the European Code Against Cancer and guidelines for quality assurance in cancer screening.
